# SF2/ASF binding region within JC virus NCCR limits early gene transcription in glial cells

**DOI:** 10.1186/1743-422X-10-147

**Published:** 2013-05-14

**Authors:** Elena Uleri, Patrick Regan, Antonina Dolei, Ilker Kudret Sariyer

**Affiliations:** 1 Department of Neuroscience, Center for Neurovirology, Temple University School of Medicine, 3500 North Broad Street, 7th Floor, Philadelphia, PA 19140, USA; 2 Section of Microbiology, Department of Biomedical Sciences, Centre of Excellence for Biotechnology Development and Biodiversity Research, University of Sassari, Sassari, Italy

**Keywords:** JC virus, PML, SF2/ASF, Replication, Ttranscription

## Abstract

**Background:**

Patients undergoing immune modulatory therapies for the treatment of autoimmune diseases such as multiple sclerosis, and individuals with an impaired-immune system, most notably AIDS patients, are in the high risk group of developing progressive multifocal leukoencephalopathy (PML), a fatal demyelinating disease of the white matter caused by human neurotropic polyomavirus, JC virus. It is now widely accepted that pathologic strains of JCV shows unique rearrangements consist of deletions and insertions within viral NCCR. While these kinds of rearrangements are related to viral tropism and pathology of the disease, their roles in molecular regulation of JCV gene expression and replication are unclear. We have previously identified SF2/ASF as a negative regulator of JCV gene expression in glial cells. This negative impact of SF2/ASF was dependent on its ability to bind a specific region mapped to the tandem repeat within viral promoter. In this report, functional role of SF2/ASF binding region in viral gene expression and replication was investigated by using deletion mutants of viral regulatory sequences.

**Results:**

The second 98-base-pair tandem repeat on Mad1 strain was first mutated by deletion and named Mad1-(1X98). In addition to this mutant, the CR3 region which served the binding side for SF2/ASF was also mutated and named Mad1-ΔCR3 (1X73). Both mutations were tested for SF2/ASF binding by ChIP assay. While SF2/ASF was associated with Mad1-WT and Mad1-(1X98), its interaction was completely abolished on Mad1-ΔCR3 (1X73) construct as expected. Surprisingly, reporter gene analysis of Mad1-(1X98) and Mad1-ΔCR3 (1X73) early promoter sequences showed two and three fold increase in promoter activities, respectively. The impact of “CR3” region on JCV propagation was also tested on the viral background. While replication of Mad1-(1X98) strain in glial cells was similar to Mad1-WT strain, propagation of Mad1-ΔCR3 (1X73) was less productive. Further analysis of the transcription mediated by Mad1-ΔCR3 (1X73) NCCR revealed that late gene expression was significantly affected.

**Conclusions:**

The results of this study reveal a differential role of CR3 region within JCV NCCR in expression of JCV early and late genes.

## Background

Replication of the neurotropic JC virus in glial cells causes the fatal demyelinating disease of the brain, progressive multifocal leukoencephalopathy (PML), which is seen in patients with underlying immunocompromised conditions, notably among AIDS patients [1-3]. PML is the only viral demyelinating disease of the human brain characterized by lytic infection of oligodendrocytes [4-6]. JCV infects greater than 80% of the human population during childhood, and establishes a latent infection in the kidneys and possibly in other body sites for the rest of the life in healthy individuals [7,8]. Recently PML has been described in patients with autoimmune diseases treated with immunomodulatory therapies. The monoclonal antibodies, natalizumab and efalizumab, are examples of these biological therapies. Natalizumab and efalizumab bind to alpha-integrin molecules on the surface of B and T cells, and prevent their entry into the brain. Another member of immunomodulatory therapy, rituximab, binds to the CD20 molecule on the surface of B cells, causing their depletion from peripheral circulation by activating the complement cascade. During the clinical trial of Tysabri, PML has been diagnosed in two multiple sclerosis patients and in one Crohn’s patient [7,9,10]. As of March 1, 2012, 212 PML cases associated with Tysabri treatment has been reported by Biogen Idec. 46 of these 212 patients with PML have died. Currently, there is no specific therapy for the treatment of PML. The non-coding control region (NCCR) of JCV is ~ 400 bp and constitutes the so called archetype form of the virus [11]. The Archetype strain of JCV is found mostly in the kidney and urine of healthy individuals. The NCCR region amplified from PML brain and cerebrospinal fluid shows unique rearrangements generated by insertions and deletions compared to the archetype strain [12], including the most studied PML type strain (Mad-1) of the virus. The NCCR of the neurotropic strain of JCV, Mad-1, is composed of two 98-bp tandem repeats that have cell type-specific characteristics. Activation of this region primarily occurs in glial cells such as oligodendrocytes and astrocytes [1,5]. Earlier studies established that cell type-specific reactivation of JCV in glial cells is primarily regulated at the transcriptional level [13]. We recently identified the alternative splicing factor, SF2/ASF, as a potential regulator of JCV whose overexpression in glial cells strongly suppresses viral gene expression and replication [14]. Unexpectedly, down-regulation of JCV by SF2/ASF is mediated at transcriptional stage, thus ascribing a novel role for SF2/ASF in the control of promoter activity. Results from a series of molecular and virological studies indicated that SF2/ASF targets the JCV promoter and strongly inhibits the JCV early and late gene transcription. Accordingly, down regulation of SF2/ASF enhances the level of viral gene expression and replication in astrocytic cells. The suppression of JCV transcription by SF2/ASF is accomplished through the interaction of SF2/ASF with a unique DNA motif within JCV promoter region (CR3 region, ~25nt in length). Among the other polyomaviruses, only the JCV promoter displays a binding motif for SF2/ASF that is conserved between archetype strain and PML-type strains (Mad1, Mad4). These observations placed SF2/ASF in a unique position in which it showed a profound ability to suppress replication and propagation of JCV by specifically interfering with viral transcription.

Here we analyzed the viral transcription and replication mediated by JCV strains which partially or completely lacked SF2/ASF binding regions (CR3) within the viral NCCR. Our results reveal a novel role of CR3 region in transcription mediated by the viral early and late promoters.

## Results

### Interaction between SF2/ASF and JCV NCCR sequences

**Figure 1:**
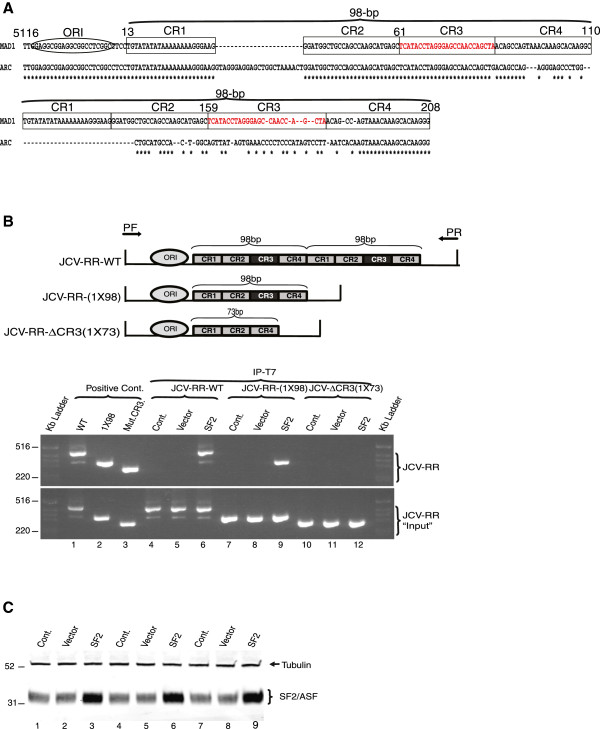
**The “CR3” region within JCV NCCR is the target for SF2/ASF. A**. Sequence alignment for the NCCR region of JCV. CLUSTAL sequence alignment was performed for JCV Mad1 and Archetype strains (gene bank accession number NC_001699). The positions of replication origin (ORI), CR1, CR2, CR3, CR4 and 98-bp-tandem repeats are illustrated in Mad1 strain. CR3 region is highlighted with sequences in red. Numbering is relative to the Mad-1 strain of JCV. **B**. Upper panel: Schematic presentation of Mad1-WT, Mad1-(1X98), and ΔCR3-(1X73) promoter. Arrows point the position of forward (PF) and reverse (PR) primers used in ChIP experiments. Lower panel: PHFA cells were transiently transfected with pCGT7-SF2/ASF expression vector (SF2) or pCGT7 vector alone (vector) and pBLCAT3-JCV-RR-WT, pBLCAT3-JCV-RR-(1X98), and pBLCAT3-JCV-RR-ΔCR3-(1X73) reporter plasmids. Cells were cross-linked and ChiP assay was performed using antibody to T7-tagged SF2/ASF (lanes 4 to 12). In lanes 1, 2 and 3, pBLCAT3-JCV-RR-WT, pBLCAT3-JCV-RR-(1X98), and pBLCAT3-JCV-RR-ΔCR3-(1X73) reporter plasmid DNAs were used as positive controls. **C**. Western blot analyzes of whole cell lysates from panel A (lanes 4 to 12), using specific antibodies against SF2/ASF and Tubulin.

Archetype strain of JC virus is mostly identified in the urine samples of healthy individuals pointing the kidneys as the potential site for latent infection [15,16]. On the other hand, archetype virus represents unique rearrangements within NCCR region that creates new pathologic strains identified in blood and CSF samples from PML patients [16-19]. The lack of archetype strain in the brain of PML patients suggests that the virus requires alterations in the NCCR region for the propagation of the virus in the glial cells. The most studied pathologic strain of JC virus (Mad1) consists of two 98-bp tandem repeats (Figure 1A). We compared sequences of Mad1 and Archetype strains using the CLUSTAL sequence alignment program. For the illustration purposes, each 98-bp tandem repeat was divided into four domains (CR1, CR2, CR3, and CR4). As shown in Figure 1A, there are multiple regions of sequence identity within the first but not in the second 98-bp tandem repeat of Mad1 and Archetype strains. Interestingly, archetype strain represents only one binding site for SF2/ASF while it was duplicated in Mad1 strain (Figure 1A, nucleotides in red, CR3 region). In order to investigate importance of the SF2/ASF binding domains, we first created a mutant NCCR construct which lacked the second 98-bp tandem repeat potentially serving only one binding site for SF2/ASF (JCV-RR-(1X98). In the second construct, in addition to the second 98-bp tandem repeat deletion, the CR3 region within the first 98-bp tandem repeat was also mutated by deletion and called JCV-RR-ΔCR3(1X73) (Figure 1B, upper panel). We first designed a series of experiments to assess the ability of SF2/ASF to bind the mutant-JCV promoter sequences. PHFA cells were transiently transfected with SF2/ASF expression plasmid and pBLCAT3 plasmid constructs containing the JCV-RR-WT, JCV-RR-(1X98), and JCV-RR-ΔCR3 (1X73) NCCR sequences and interaction of SF2/ASF with mutant viral sequences were analyzed by ChIP assay as described in materials and methods. As expected, ChIP analysis of PHFA cells demonstrated association of SF2/ASF with JCV-RR-WT and JCV-RR-(1X98) mutant promoter sequences (Figure 1B). Meanwhile SF2/ASF showed no interaction with JCV-RR-ΔCR3 (1X73) promoter (Figure 1B, lane 12) confirming that CR3 region was mainly the site where SF2/ASF associated [14].

### The second 98-bp tandem repeat and “CR3” region limit JCV-early gene transcription

**Figure 2:**
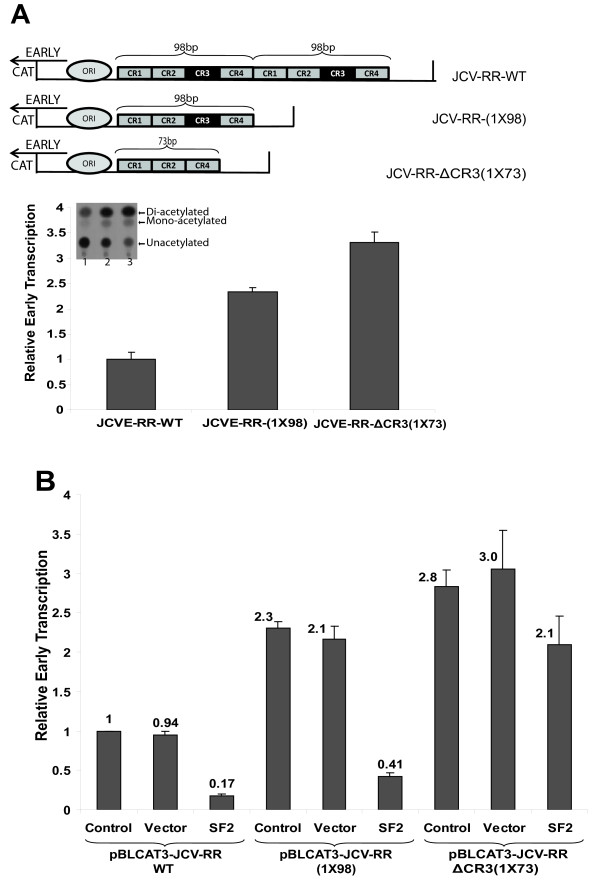
**Transcriptional activities of mutant JCV promoter sequences. A**. Cat enzyme activity of JCV-early promoter constructs were detected, and presented as bar graph. Schematic representation of JCV NCCR sequences cloned into CAT reporter constructs in early orientations was shown at the top of the graph. **B**. Effect of SF2/ASF on transcription induced by mutant JCV promoter sequences. pBLCAT3-JCVE-RR WT, pBLCAT3-JCVE-RR-(1X98), and pBLCAT3-JCVE-RR- ΔCR3-(1X73) reporter plasmids were transiently transfected into PHFA cells in the presence or absence of either pCGT7 vector alone or pCGT7-SF2/ASF expression plasmid. Cat enzyme activity of JCV promoter constructs were detected, and presented as bar graph.

To determine the transcription mediated by JCV-early promoter sequences from Mad1-WT, Mad1-(1X98), and Mad1-ΔCR3 (1X73), promoter sequences were cloned into CAT reporter plasmids. PHFA cells were transiently transfected with these constructs and basal transcriptional activities were determined by reporter gene assays as described in the materials and methods. As shown in Figure 2A, the reporter construct with one copy of 98-bp tandem repeat showed two fold higher transcriptional activity than WT construct. More interestingly, the third reporter construct [JCVE-RR-ΔCR3 (1X73)] showed significantly higher transcriptional activities than both JCVE-RR-(1X98) and JCVE-RR-WT. These results suggested that the second 98-bp tandem repeat and CR3 region within JCV promoter limited the basal transcription mediated by JCV-early promoter. Next, we analyzed the effect of SF2/ASF on early gene transcriptional activity mediated by mutant JCV-promoter sequences. As expected, SF2/ASF suppressed transcription induced by the wild type and the mutant [pJCV-RR-(1X98)] promoters (Figure 2B, compare control, vector, and SF2 bars). As hypothesized, SF2/ASF did not show any significant suppression on transcription mediated by the JCV- ΔCR3 (1X73) promoter, which lacked both “CR3” regions. These results suggest that SF2/ASF binding to the JCV promoter through the CR3 region might be crucial for the suppression of viral early gene transcription.

### Propagation of JCV- Mad1 (1X98) and JCV-Mad1-ΔCR3 (1X73) strains in PHFA cells

**Figure 3:**
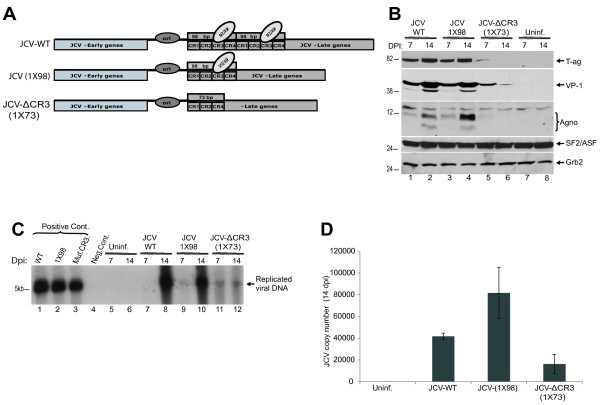
**Viral propagation of mutant JCV strains in PHFA cells. A**. Schematic representation of JCV wild type and mutant genomes. **B**. Western blot analyzes of whole cell extracts from PHFA cells infected with JCV-Mad1-WT and mutants, JCV-Mad1-(1x98), and JCV-Mad1-ΔCR3-(1X73), using specific antibodies against LT-Ag, VP1, SF2/ASF and Agno protein. In lanes 7 and 8, whole cell extracts from uninfected cells were loaded as negative control. Western blot analyzes of same extracts with anti-Grb2 antibody was used as loading control. DPI depicts “day post-infection”. **C**. Southern blot analyses of replicated JCV genomic DNAs in parallel to protein samples in panel **A**. In lanes 1, 2 and 3, 2 ng of linearized Mad1-WT, Mad1-(1X98), and Mad1-ΔCR3-(1X73) were used as positive controls, respectively. In lane 4, DNA samples from uninfected cells were loaded as negative control. **D**. Q-PCR analyses of the JCV copy numbers in growth media of the infected PHFA cultures. Culture media was collected at 14 dpi, and was processed for the detection of viral particles by Q-PCR as described earlier [12,16].

Reporter gene analyses of mutant JCV promoter sequences (Figure 2A) suggested that CR3 region of JCV-NCCR limits the early gene transcription of the virus. However, these experiments were performed in the absence of viral early and late genes which might also influence the activity of viral transcription induced by wild type and mutant viral constructs. In order to test the possible impact of “CR3” region on viral propagation, same promoter mutations in the reporter constructs (Figure 2A) were also created on viral background. PHFA cells were transfected/infected with these viral genomes as described in materials and methods and whole cell extracts were analyzed for the expression of viral early (LT-Ag) and late genes (VP-1 and Agno) proteins at 7 and 14 days post-infections. As shown in Figure 3B, the level of LT-Ag, VP-1 and Agno protein expressions from the Mad1-WT and Mad1-(1X98) gradually increased as the JCV infection cycle reached 14d post-infection (lanes 1–4). Surprisingly, the level of VP-1 expression from the Mad1-ΔCR3(1X73) started markedly lower than Mad1-WT and Mad1-1X98 at 7d post-infections, and decreased dramatically at 14d post-infections (Figure 3B, compare lanes 5–6 with lanes 1–4). On the other hand, expression levels of LT-Ag and Agno protein was undetectable in cellular extracts prepared from the cells infected with the Mad1-ΔCR3(1X73) at day 14, and barely detectable for 7d post-infection (Figure 3B, middle panel, lanes 5–6). Since viral gene expression is negatively affected in the Mad1-ΔCR3 (1X73) infections, we next assessed the functional consequences of this effect on viral DNA replication by employing a Dpn I replication assay in parallel to western blot analysis of viral genes. Subsequently, newly replicated Dpn I- resistant DNA was analyzed by Southern blotting. It was observed that the replication efficiency of the Mad1-ΔCR3 (1X73) was much lower than Mad1-WT and Mad1-(1X98) (Figure 3C). JCV particles in growth medium of infected cells were also analyzed and quantified by Q-PCR analysis at 14d post-infections. As shown in Figure 3D Mad1-ΔCR3-(1X73) showed significantly lower levels of viral copies in growth media as compared with Mad1-WT and Mad1-(1X98).

### JCV-Mad1-Mut.CR3-(1X73) is defective in late gene transcription

**Figure 4:**
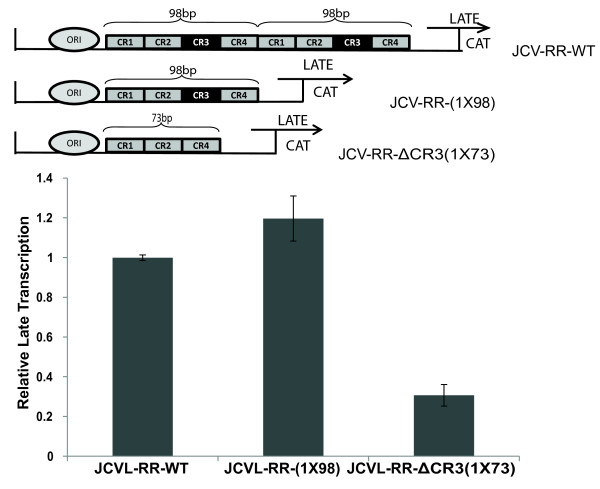
**The “CR3” region within JCV NCCR is critical for the expression of late genes.** Cat enzyme activity of JCV-late promoter constructs were detected, and presented as bar graph. Schematic representation of JCV NCCR sequences cloned into CAT reporter constructs in late orientations was shown at the top of the graph.

Mutational analyses of the second 98-bp tandem repeat and CR3 region within JCV promoter suggested that JCV-Mad1- ΔCR3(1X73) showed two and three fold higher transcriptional activity for the early genes than JCV-Mad1-(1X98) and JCV-Mad1-WT, respectively (Figure 1B). Unexpectedly, propagation of JCV-Mad1- ΔCR3-(1X73) was insufficient in PHFA cells (Figure 3). JCV-NCCR is a bi-directional promoter which simultaneously encodes early and late genes. The observed defect in replication of JCV-Mad1- ΔCR3-(1X73) suggested that late gene expression could be affected by the applied mutations within the viral promoter. To determine the transcription mediated by JCV-late promoters, Mad1-WT, Mad1-(1X98), and Mad1- ΔCR3 (1X73) NCCRs were cloned into a CAT reporter construct in late orientations (Figure 4A). PHFA cells were transiently transfected with these constructs and basal transcriptional activities were determined by CAT assay. As shown in Figure 4, the Mad1-WT and Mad1-(1X98) showed comparable transcriptional activities. However, the third reporter construct with no CR3 region [Mad1- ΔCR3 (1X73)] showed significantly lower transcriptional activity than Mad1 WT and Mad1-(1X98). These results suggest that the “CR3” domain within the first 98-bp tandem repeat was critical for the expression of viral late genes.

## Discussion

JCV infects human population in childhood and establishes a persistent latent infection for the rest of the life. The JCV genome detected in urine of healthy individuals shows a linear noncoding control region called archetype NCCR. On the other hand, the JCV genome in CFS samples from PML patients show rearranged NCCRs involving deletions and insertions within this region, suggesting that the archetype strain of the JCV is not able to propagate in the brain without the proper rearrangements [18,19]. While these kinds of rearrangements are related to viral tropism and pathology of the disease, their roles in molecular regulation of the JCV gene expression and replication in glial cells are unclear. Our results in this report expand and confirm our previous report showing SF2/ASF, a cellular alternative splicing factor, targets a unique sequence within JCV NCCR and strongly inhibits viral transcription and viral propagation in glial cells [14]. Here we created mutant viral strains, which partially or totally missing SF2/ASF binding domains, on the Mad1 strain, a rearranged viral strain isolated from a patient’s brain with PML which consists of two 98-bp tandem repeats within the viral NCCR and contains two binding sites for SF2/ASF. In the first construct we deleted the second 98-bp repeat and investigated the impact of this large deletion on viral transcription and replication by reporter gene analysis and viral propagation assays. While the Mad1 genome with only one 98-bp tandem repeat showed more than two fold higher early transcriptional activity than wild type promoter, it only showed a slight increase in the late gene transcriptional activity. The mutant strain with only one 98-bp tandem repeat is highly relevant to the clinic since there have been same viral strains isolated from patients with colon cancer. Interestingly, JCV Mad1 strain was isolated in colon cancer tissue, and the variant with 98-bp tandem repeat deletion was exclusively found in these cancers [20]. Our results suggest that deletion of one 98-bp tandem repeat within the Mad1 NCCR increases the rate of early gene transcription which express viral tumor antigens, large T-Ag and small t-ag, and may suggest a possible mechanism for the involvement of JCV in the formation of colon cancers. Whether the Mad1 strain with one 98-bp tandem repeat will show higher early gene expression in colon cancer cells needs to be clarified and possible involvement of this strain in human colon cancer development needs to be further investigated. In addition to deletion of the second 98-bp tandem repeat, we also created a viral construct with no binding site for SF2/ASF [JCV-Mad1-ΔCR3 (1X73)] in which the CR3 region within the first 98-bp repeat was also mutated by deletion. While early transcription was significantly increased by the Mad1-ΔCR3 (1X73) NCCR, there was a dramatic decrease in the levels of viral replication. Further analyses of the transcription mediated by Mad1-ΔCR3 (1X73) NCCR revealed that late gene expression was significantly affected. This might explain the less productive viral propagation initiated by JCV-Mad1-ΔCR3 (1X73) in glial cells. One could argue that removal of the CR3 region along with second 98-bp tandem repeat would disrupt the transcriptional initiation for the late genes. Western blot analysis of the protein extracts from glial cells infected with Mad1-ΔCR3 (1X73) revealed that major capsid protein VP1 expression was detectible at 7 day post infections suggesting that late gene transcription was successfully initiated. However, protein expression levels were much lower than either Mad1-WT or JCV-Mad1-(1X98). One possible explanation for the decreased levels of late gene expression could be the transcription factors which bind to the CR3 region to enhance the late gene transcription. This region encompasses predicted binding sites for several transcription factors including Purα, NF-1, MF3, Elk-1, COE1, and p300. The importance of these transcription factors and their interaction with SF2/ASF for the regulation of JCV late gene transcription remains to be investigated. It was surprising to observe that LT-antigen expression was reduced in cells infected with JCV-Mad1-ΔCR3 (1X73), because of the early promoter activity of this viral strain was much higher than Mad1-WT and Mad1-(1X98). One possible explanation of the reduced levels of LT-ag expression could be due to the autoregulation of the protein. LT-antigens of polyomaviruses are well known as auto-regulatory proteins [21] and viral NCCR sequences have shown to be important for the autoregulation of the protein [22,23], Low levels of LT-ag expression by JCV-Mad1-ΔCR3 (1X73) virus at 7 dpi infection could be due to the alteration of this autoregulation. Alternatively, our data indicated that JCV-Mad1-ΔCR3 (1X73) construct was less efficient for the late gene transcription. The observed low levels of LT-ag could also be due to the inefficient late gene transcription since that was resulted in a dramatic decrease of viral propagation.

## Conclusions

The aim of this study was to investigate the role of SF2/ASF binding region in JC virus gene expression and replication by using deletion mutants of viral regulatory sequences. Reporter gene analyses of Mad1 promoter sequences with mutations either partially or totally lacking SF2/ASF binding regions showed a significant increase in early promoter activities suggesting that SF2/ASF binding region (CR3) put a negative pressure on viral early transcription. Following results from viral propagation and reporter gene studies suggested that the CR3 region was crucial for the propagation of the virus and the transcription of late genes in glial cells. The results of this study suggest a novel role of the second 98-bp tandem repeat and CR3 region within the first 98-bp-repeat of JCV promoter in transcription mediated by the viral early and late promoters.

## Methods

### JCV strains and plasmid constructs

The wild type Mad1 strain of JCV was linearized by BamH1 digestion, and cloned into the pBlueScript KS (+) vector previously [14]. To mutate the second 98 bp repeated region on JCV promoter, the pBlue-Mad1-WT construct was digested with Avr II restriction enzyme which cut viral genome once within 98 bp repeat regions. The digested product was gel purified, re-ligated, sequenced, and named as pBlue-Mad1-(1X98). The pBlue- JCV-Mad1-ΔCR3(1X73) construct which lacked the CR3 region within the 98-bp-repeat, was created from pBlue-Mad1-(1X98) template by using following primers: F.JCV-RR-(86–118); 5’-ACAGCCAGTAAACAAAGCACAAGGGGAAGTGGA-3’, R.JCV-RR-(60–28); 5’-GCTCATGCTTGGCTGGCAGCCATCCCTTCCCTT-3’. The amplified product was gel purified, ligated, and sequenced. pBLCAT3-JCV-RR-WT reporter construct was described previously [24]. Reporter gene constructs containing the regulatory region of the JCV Mad1-(1X98) and JCV-Mad1- ΔCR3 (1X73) were created as follows. Mad-1 (4987–240) region was PCR-amplified using appropriate primers from Mad1-(1X98) and Mad1- ΔCR3 (1X73) templates and was inserted into Bam HI site of pBLCAT3 vector in early (E) and late (L) orientations. The resulting reporter plasmids were called pBLCAT3-JCV-RR-(1X98) and pBLCAT3-JCV-ΔCR3(1X73. pCGT7-SF2/ASF-FL expression plasmid was a kind gift from Dr. Javier F. Ca’ceres (Medical Research Council Human Genetics Unit, Western General Hospital, Edinburgh EH4 2XU, Scotland, United Kingdom).

### Cell lines

Primary human fetal astrocytic cells (PHFA) were prepared as previously described [25]. Briefly, human fetal brain tissue was obtained from Advanced Biosciences Resources, Inc. (Alameda, CA). The tissue was washed with HBSS medium and placed in a 100 mm dish. Blood vessels and meninges were dissected, and tissue was cut into small pieces using a forceps and scalpel. Chopped-tissue was mechanically disrupted by pipetting up and down in HBSS with a 10 ml pipette until cell culture fluid smooth and pinkish in color. The tissue was centrifuged and digested with DNAse I and trypsin in 10 ml HBSS medium for 30 minutes at 37 0C. Cells were washed with HBSS and passed through a 70-micron filter. Mixed cultures of glial cells were plated in Poly-D-Lysinized T162 cm 2 flasks with DMEM/F12 medium (1/1) containing 10% FBS, 1% L-glutamine, 1% Fungizone, insulin, and gentamycin. After plating 4–5 days, the cells were washed with PBS and trypsinized. They were then plated in T162 cm 2 flasks and incubated for 45 minutes. During the 45 minute period, microglial cells attached to the flask, and most of the astrocytes, neurons, and oligodendrocytes remained in the medium. After 45 minute incubation, the medium was removed and placed in new flasks. The cells were grown in culture until they were confluent. Once confluent, the cells were placed on an orbital shaker to remove the neurons and oligodendrocytes, which detached from the surface of the flasks and came off into the medium. After proper shaking, the medium was replaced with astrocyte growth medium, DMEM/F12 (1/1) with 15% FBS, 1% L-glutamin, insulin, and gentamycin.

### ChIP assay

PHFA cells were transiently transfected with pBLCAT3-JCV_E_-RR-WT, pBLCAT3-JCV_E_-RR-(1X98), and pBLCAT3-JCV_E_-RR-(Mut.CR3) reporter constructs in the presence or absence of pCGT7-SF2/ASF expression plasmid. ChIP assays were performed at 72 h post-transfections as described previously [14]. Briefly, proteins were cross-linked to DNA by formaldehyde, following by sonication to fragment the chromatin and immunoprecipitation of specific proteins to obtain DNA segments. Cross-linking was reversed and DNA was analyzed by PCR.

### Transcription assay

Chloramphenicol acetyltransferase (CAT) assay was performed as described before [14,24]. Briefly, PHFA cells were plated in 60 mm tissue culture dishes and transiently transfected with pBLCAT3-JCV-RR-WT, pBLCAT3-JCV-RR-(1X98), and pBLCAT3-JCV-RR-ΔCR3 (1X73) reporter constructs in the presence or absence of pCGT7-SF2/ASF expression plasmid. At 48 h post-transfection, cells were extracted with a series of freeze/thaw cycles, and the CAT activity of the samples was determined.

### JCV infection

Transfection/infection of cells with the full-length JCV Mad-1 genome was described previously [14,24,26]. Briefly, pBlue-Mad1-WT pBlue-Mad1-(1X98) and pBlue-JCV-Mad1-ΔCR3 (1X73) were digested with BamH1 enzyme to remove the complete viral genomes from pBlue.Script KS (+) plasmid. PHFA cells, at a confluence of 1 × 10^6^ cells per T75-cm tissue culture flask, were co-transfected/infected with the JCV-Mad1-WT, JCV-Mad1-(1X98), and JCV-Mad1-(Mut.CR3) DNA (10 μg/flask) using Fugene6 transfection as indicated by the manufacturer (Roche). At 12 days post-infection, cells were trypsinized and split into two equal portions. One half was used for preparation of whole cell protein extract for Western blot analysis, and the other half was used for DNA preparation using Qiaprep Spin Miniprep kit (Qiagen).

### Southern blotting

Replication assays were carried out as previously described [14,24]. Briefly, PHFA cells (1 × 10^6^ cell/75 cm^2^ flask) were transfected/infected as described above. Low molecular weight DNA purified from JCV-infected cells was digested with Dpn I and BamH1 enzymes [27]. Digested-DNA samples were separated on 1% agarose gel and were transferred to a nylon membrane. Replicated viral DNA was visualized upon incubation of the membrane with a [^32^P]-labeled JCV DNA probe as described earlier [14].

### Quantitative-PCR (Q-PCR) analyses of JCV copy numbers in growth media

Transfection/infection of cells with the full-length JCV-Mad1 genome was performed as described above. The culture medium (containing viral particles) was collected at 12 days post-infection, and after centrifugation at 13,000 rpm for 10 minutes to remove cell debris, supernatants were collected and incubated at 95°C for 10 minutes to inactivate virus. Ten microliters of the medium was then used as a template in Q-PCR reactions. The standard curve was obtained after serial dilution of pJCV, a plasmid containing the whole genome of the JCV Mad-1 strain. The standard curve was then used to extrapolate the viral load of each sample. Negative and positive controls were included in each reaction and each sample was tested in triplicate. All Q-PCR analyses were done by using Light cycler 480 (Roche). Primers were JCV Q-PCR-forward: 5’-AGTTGATGGGCAGCCTATGTA-3’ and JCV Q-PCR-reverse: 5’- TCATGTCTGGGTCCCCTGGA-3’. The probe for the Q-PCR was 5’-/5HEX/CATGGA TGCTCAAGTAGAGGAGGTTAGAGTTT/3BHQ_1/-3’.

## Competing interests

The authors declare that they have no competing interests.

## Authors’ contribution

Conceived and designed the experiments: IKS. Performed the experiments: EU, PR, and IKS. Analyzed the data: IKS, EU and AD. Wrote the paper: IKS. All authors read and approved the final manuscript.
